# Enhanced Plasmonic Biosensor Utilizing Paired Antibody and Label-Free Fe_3_O_4_ Nanoparticles for Highly Sensitive and Selective Detection of Parkinson’s *α*-Synuclein in Serum

**DOI:** 10.3390/bios11100402

**Published:** 2021-10-18

**Authors:** Samuel Husin Surya Mandala, Tai-Jan Liu, Chiung-Mei Chen, Kuo-Kang Liu, Mochamad Januar, Ying-Feng Chang, Chao-Sung Lai, Kuo-Hsuan Chang, Kou-Chen Liu

**Affiliations:** 1 Department of Electronic Engineering, Chang Gung University, Taoyuan 33302, Taiwan; samuelhusin31@gmail.com (S.H.S.M.); moch.januar@outlook.com (M.J.); cslai@mail.cgu.edu.tw (C.-S.L.); 2 Department of Traditional Chinese Medicine, Chang Gung Memorial Hospital, Keelung 204, Taiwan; sarah78912@hotmail.com; 3 Department of Neurology, Chang Gung Memorial Hospital, Chang Gung University College of Medicine, Taoyuan 33302, Taiwan; cmchen@cgmh.org.tw; 4 School of Engineering, University of Warwick, Coventry CV4 7AL, UK; I.K.Liu@warwick.ac.uk; 5 Artificial Intelligence Research Center, Chang Gung University, Taoyuan 33302, Taiwan; yfchang@mail.cgu.edu.tw; 6 Biosensor Group, Biomedical Engineering Research Center, Chang Gung University, Taoyuan 33302, Taiwan; 7 Department of Nephrology, Chang Gung Memorial Hospital, Linkou, New Taipei City 33305, Taiwan; 8 Department of Materials Engineering, Ming Chi University of Technology, New Taipei City 24301, Taiwan; 9 Department of Neurology, Chang Gung Memorial Hospital Linkou Medical Center and College of Medicine, Chang Gung University, Linkou 33305, Taiwan; gophy5128@cgmh.org.tw; 10 Division of Pediatric Infectious Disease, Department of Pediatrics, Chang Gung Memorial Hospital, Linkou 33305, Taiwan

**Keywords:** α-synuclein, Parkinson’s disease, surface plasmon resonance, Fe_3_O_4_ nanoparticles, paired antibody, human serum

## Abstract

Parkinson’s disease (PD) is an acute and progressive neurodegenerative disorder, and diagnosis of the disease at its earliest stage is of paramount importance to improve the life expectancy of patients. α-Synuclein (α-syn) is a potential biomarker for the early diagnosis of PD, and there is a great need to develop a biosensing platform that precisely detects α-syn in human body fluids. Herein, we developed a surface plasmon resonance (SPR) biosensor based on the label-free iron oxide nanoparticles (Fe_3_O_4_ NPs) and paired antibody for the highly sensitive and selective detection of α-syn in serum samples. The sensitivity of the SPR platform is enhanced significantly by directly depositing Fe_3_O_4_ NPs on the Au surface at a high density to increase the decay length of the evanescent field on the Au film. Moreover, the utilization of rabbit-type monoclonal antibody (α-syn-RmAb) immobilized on Au films allows the SPR platform to have a high affinity-selectivity binding performance compared to mouse-type monoclonal antibodies as a common bioreceptor for capturing α-syn molecules. As a result, the current platform has a detection limit of 5.6 fg/mL, which is 20,000-fold lower than that of commercial ELISA. The improved sensor chip can also be easily regenerated to repeat the α-syn measurement with the same sensitivity. Furthermore, the SPR sensor was applied to the direct analysis of α-syn in serum samples. By using a format of paired α-syn-RmAb, the SPR sensor provides a recovery rate in the range from 94.5% to 104.3% to detect the α-syn in diluted serum samples precisely. This work demonstrates a highly sensitive and selective quantification approach to detect α-syn in human biofluids and paves the way for the future development in the early diagnosis of PD.

## 1. Introduction

The circulation of disease biomarkers in the human body, including DNA/RNAs, proteins, enzymes, and metabolites, has great potentials for clinical diagnosis, prognostic treatment, and monitor disease progression [1]. In general, the altered level of the biomarker concentrations in human body fluids have been used as diagnostic indicators of cancer [2], autoimmune [3], and neurodegenerative disorders [4]. Parkinson’s disease (PD) has been conceded as the second most common neurodegeneration disease after Alzheimer’s disease, affecting more than 1% of the global population, with an incident rate likely to double the increase in 2030 [5]. The clinical diagnosis of PD is mainly based on the manifestation of patient’s movement difficulties, such as bradykinesia, stiffness, tremor, and slowness [6]. However, when the patient meets the clinical assessment of PD, almost 60–70% of the dopaminergic neurons in the substantia nigra are lost [7,8]. Treatment can only be initiated after the late phase of the disease, which causes the disease-modifying drugs not efficiently reversing or stopping the disease progression [9]. Therefore, detecting levels of specific biomarkers for the diagnosis and prognosis of PD could be beneficial to identify the patients at the earlier disease stages; thus, the treatment can be expedited before the clinical onset occurs, which might increase the life expectations of patients.

Selecting specific biomarkers of PD and subsequently detected by using an affordable and reliable analytical platform is one strategy to implement early diagnosis of PD. α-Synuclein (α-syn), which constitutes a protein with molecular weight of 14 kDa and encoded by the SNCA gene, has been emerging as a potential biomarker for the non-invasive diagnosis of PD clinically [10,11]. The aberrant aggregation of α-syn to form Lewy bodies and Lewy neurites in neuronal substantia nigra has been identified as a major factor in the pathogenesis of PD [12]. Extensive research has shown that the aberrant level of α-syn can be detected in human body fluids, such as peripheral blood [13]. In this regard, several reports have shown that elevated levels of α-syn have been detected in the clinical blood plasma and serum for PD patients compare to healthy individuals [14,15,16]. Thus, detecting the increased level of α-syn from the blood of PD patients can help to understand the progression of the disease, which is essential for its early diagnosis. For such detection, a wide range of analytical platforms, including enzyme linked immunosorbent assays (ELISA) [15], electrochemical immunosensors [17], fluorescent immunoassay [18], immunomagnetic reduction (IMR) [14], and neurobiosensors [19], has, therefore, been developed. These methods are generally feasible in the laboratory but less achievable in clinical practice due to their difficulty in amplification, expensive analytical reagent, long period measurement, and complex operations. Surface plasmon resonance (SPR) biosensors have been known as a well-established platform for monitoring biomolecular interactions at sensor interfaces [20]. The merits of SPR over the common platforms for PD diagnosis include label-free approach, high reproducibility, real time measurement, and simple operations [21], which are desirable for developing the affordable point of care in clinical PD diagnosis. In general, using traditional SPR techniques is limited to detect the analyte with a concentration lower than 1 pM (corresponded to 8 kDa molecular weight) due to the high attenuation wave on the thin Au film [22,23]. The intrinsic characteristics of the SPR sensor fails to meet the required sensitivity for the clinical diagnosis of PD because the α-syn concentration in PD patients can be very shallow, as low as 3.60 ± 2.53 pg/mL in plasma and 0.031 ± 0.04 pg/mL in serum [14]. More seriously, one of the drawbacks is its selectivity for the biomarker detection in blood samples because any non-specific binding (NSB) of proteins onto the sensor surface can generate a false SPR signal which cannot be discriminated from the target analyte [24]. Therefore, the development of an improved SPR platform for α-syn detection at the femtogram level with high selectivity in a blood sample is critical for clinical PD diagnosis.

Currently, there are two promising ways to improve the selectivity of the SPR platform and amplify the detection of α-syn in a blood sample: (1) incorporating a pair of bioreceptor, such as antibody–antibody for a target protein; and (2) immobilizing metal nanoparticles, such as iron oxide nanoparticles (Fe_3_O_4_ NPs), on the metal surface. For the first approach, utilizing a pair of bioreceptors has been demonstrated as an effective strategy to reduce the effect of NSB in complex solutions [25,26,27]. By adding a secondary bioreceptor to pair with a capture bioreceptor, the level of background effect from the secondary bioreceptor can be minimized after injecting the analyte in a biofluid [21]. As a result, quantitation of the analyte is then possible in complex biofluids. For the second approach, metal nanoparticles have attracted a lot of attention in the scientific community because they are superior to enhancing the SPR sensitivity [22,28]. For instance, utilizing the considerable size and high refractive index of Fe_3_O_4_ NPs on the gold (Au) metal film can increase the local refractive index of the Au surface; thus, increasing the sensitivity of the SPR platform from 1300 nm/RIU to 5200 nm/RIU [29]. In addition, the Fe_3_O_4_ NPs has low optical loss compared to the Au nanoparticles (AuNPs), as a standard method for enhancing SPR sensitivity [30]. As a result, Fe_3_O_4_ NPs can be immobilized on the Au surface with high density nanoparticles to support a maximum sensitivity enhancement without suffering from the high optical loss due to the scattering effect from denser nanoparticles [30,31]. However, several previous studies have reported the use of Fe_3_O_4_ NP as a labeling tag, which can expense the detection procedure, increase the complexity of the assay, and reduce the colloidal stability in a complex medium [21,29]. Up to now, no studies have reported the utilization of the Fe_3_O_4_ NPs in SPR sensor via label-free structure to detect α-syn biomarkers in a serum sample. Therefore, label-free Fe_3_O_4_ NPs in combination with a pair of bioreceptor is promising to detect low-level α-syn in the serum samples because they can increase the SPR sensitivity and enhance detection selectivity for rapid and massive testing.

**Figure 1:**
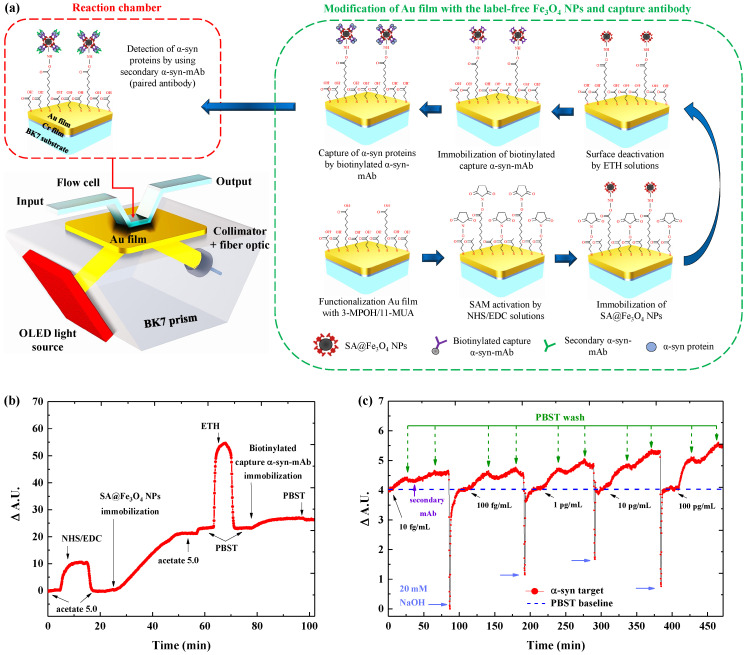
(**a**) Schematic configuration of the SPR sensor integrated with the OLED light source. Inset: the process of the surface modification and α-syn detection based on the label-free SA@Fe_3_O_4_ NPs structure and antibody pairs on the Au film surface (the green-color box). (**b**) SPR sensorgram for sensor surface modification and (**c**) detection of α-syn protein at various concentration using pair of antibodies.

In this paper, we report the first application of label-free Fe_3_O_4_ NPs in combination with pair of antibodies integrated to the Au metal surface for the detection of α-syn in serum samples based on real-time SPR analysis. An illustration of the surface structure incorporated with the proposed platform is shown in Figure 1a. We have investigated the effect of Fe_3_O_4_ NPs assembly on the Au sensing surface to enhance the SPR sensitivity by varying the dilution factor of nanoparticles. The binding affinity and selectivity of two different types of animal α-syn antibodies (rabbit host and mouse host) have also been observed to select a more suitable type used for detecting α-syn in the serum sample. Finally, our label-free Fe_3_O_4_ NPs with pair of antibody–antibody were applied for direct measurements of α-syn concentrations in diluted serum samples, which can trace the biomolecules until femtogram level detection with low background signals. This proposed methodology holds intriguing potential for developing SPR platforms with highly sensitive and selective detection on α-syn in a serum sample, which can be further applied for clinical PD diagnosis purposes.

## 2. Experimental

### 2.1. Materials

Rabbit anti-α-syn monoclonal antibody (α-syn-RmAb, Ab138501), mouse anti-α-syn monoclonal antibody (α-syn-MmAb, Ab1903), and rapid biotinylation kit were purchased from Abcam Inc. For the capture antibody, α-syn-RmAb and α-syn-MmAb were functionalized with biotin using a rapid biotinylation kit following the manufacturing procedure. Phosphate buffer saline (PBS, pH 7.4), immunoglobulin (IgG), surfactant tween-20, bovine serum albumin (BSA), ethanol, human AB male serum, 3-mercapto-1-propanol (3-MPOH), and 11-mercaptoundecanoid (11-MUA) were supplied by Sigma-Aldrich. The 0.01 M sodium acetate buffer solution, the regeneration buffer solution (20 mM NaOH and glycerin pH 2.0), N-hydroxysuccimide (NHS), 1-ethyl-3 (3-dimethylaminopropyl)carbodiimide (EDC), and ethanolamine-hydrochloride solution (ETH, pH 8.5) were purchased from Cytiva. Human recombinant α-syn was purchased from BostonBiochem. Streptavidin-conjugated Fe_3_O_4_ (SA@Fe_3_O_4_ NPs) was supplied by Ocean NanoTech. All reagents were of analytical grade and used without further purification, and ultra-pure distilled water (DI water) was used throughout the experiments.

### 2.2. Apparatus

The configuration of the portable SPR device is illustrated in Figure 1a. The light source of the SPR system was supported by an organic light-emitting diode (OLED) with a peak wavelength of 610 nm (purchased from Ultimate Image Corp., Hsinchu City, Taiwan). The OLED panel was integrated with a brightness-enhanced film (BEF) and a giant-birefringent-optical film (GBO) [32]. The light emitted by the integrated OLED panel was passed through the BK7 prism at an angle of incidence of 75°, which was then reflected by the sensor chip (Au film). The reflected light was received by the collimator and transmitted to the spectrometer (Ocean Optics, FL, USA) *via* optical fiber to be converted into an SPR signal. The measured SPR signal was normalized to the SPR signal of the NHS/EDC sample (Δ A.U.).

The shape and size of SAFe_3_O_4_ NPs prepared for this study were characterized by a tunneling electron micrograph (TEM) (JEOL JEM-2100 Plus, Tokyo, Japan). X-ray diffraction (XRD) patterns of SA@Fe_3_O_4_ NPs were measured on Bruker D8 Discover system. The density of immobilized nanoparticle on the sensor surface was recorded by using scanning electron microscope equipped with a field emission gun (FE-SEM, JEOL JSM-7500F operating at 5 kV). The nanoparticle density was calculated from the average of the FE-SEM images recorded from four different sample regions (14.8 μm^2^).

### 2.3. Functionalization of Sensor Chips

The SPR sensor chips were fabricated by depositing 47 nm of Au films and 3 nm of chromium film (Cr) on BK7 glass substrate by using thermal evaporation. The fabricated sensor chips were modified with a mixed self-assembled monolayer (SAM) consisting of 10 mM 3-MPOH and 1 mM 11-MUA at a mixing ratio 10:1 in ethanol solutions. The sensor chips were immersed in the mixed SAM solution overnight in order to generate an optimized thiol binding matrix on the Au sensor surface.

In order to enhance the SPR platform detecting α-syn proteins, the amine coupling procedure was performed to produce the structure of label-free SA@Fe_3_O_4_ NPs and the capture antibodies on the Au film surface, which illustrates in Figure 1a (green-color box). Firstly, the thiol layers on Au film surface were activated by using a mixture solution of NHS/EDC (1:1 DI water of 0.1 M NHS and 0.4 mM EDC) for 10 min, followed by rinsing with the acetate solution for 8 min. Secondly, the SA@Fe_3_O_4_ NPs were diluted in 1 mL volume of acetate buffer with a dilution factor of 1:100, 1:20, and 1:5. Then, the diluted SA@Fe_3_O_4_ NPs were dispensed onto the activated sensor surface for about 40 min. Subsequently, the acetate solution was injected for 8 min to wash the excess SA@Fe_3_O_4_ NPs on the sensor surface. The third step is to deactivate the reacted sites, which are not covalently bond with the SA@Fe_3_O_4_ NPs, by injecting an ETH solution for 8 min and followed by washing with PBST buffer (consisted 0.1 M PBS buffer + 0.005% (v/v) tween-20) for 10 min. Finally, 20 μg/mL biotinylated monoclonal antibody (α-syn-RmAb or α-syn-MmAb) was immobilized to SA@Fe_3_O_4_ NPs *via* the avidin-biotin interaction for 40 min, and the PBST buffer flowed for 10 min. The surface modification procedures were recorded by SPR sensorgram as presented in Figure 1b.

### 2.4. Detection Protocol of Immunoassays

For measuring α-syn in the biological environment, PBST buffer was prepared as a biological solution because the addition of tween-20 into PBS buffer can reduce the NSB effect during the subsequent experiment. The various concentrations of α-syn proteins (10 fg/mL to 100 pg/mL) were prepared in 1 mL volume of PBST buffer. Subsequently, the prepared α-syn sample was injected into the reaction chamber and the hybridization between the analyte and the immobilized antibody was monitored for approximately 20 min. To remove the excess α-syn in the reaction chamber, the sensor surface was rinsed with PBST solution for 8 min. The signal for the infused α-syn sample was measured based on the signal difference between the post-washed sample and the pre-infused α-syn sample. Next, the secondary anti-α-syn-mAb at the concentration of 1 ng/mL in PBST buffer was immobilized onto the sensor surface to interact with the captured α-syn proteins for 20 min, and then washed by flowing PBST buffer for 8 min. The SPR signal for the binding of the secondary anti-α-syn-mAb with the captured α-syn could be determined based on the signal difference of the SPR sensorgram after the binding and washing process on the secondary antibody sample. Finally, the sensor surface was restored using the regeneration solution for 1 min and washed with PBST buffer until the SPR signal reached a stable baseline. The signal of this process was recorded by SPR sensorgram and shown in Figure 1c.

For the measurement of α-syn in serum samples, the human AB male serum was diluted 1000 times in PBST buffer. After that, the various concentration of α-syn protein ( 10 fg/mL to 10 pg/mL) was added into the diluted serum. The detection procedure in the diluted serum samples was the same as that in the PBST buffer.

### 2.5. Specificity Test

To assess the selectivity of the sensing platform, the un-specific proteins in serum (BSA and IgG with 1 μg/mL concentration of each substance) and the target protein (α-syn protein with 100 fg/mL concentration) were monitored using the aforementioned new platform.

## 3. Results

### 3.1. Influence of Fe_3_O_4_ NPs Density on the SPR Sensitivity

The shape, size, and structure of SA@Fe_3_O_4_ NPs were characterized by using TEM images and XRD measurement. Figure S1a shows the TEM images of a number of the SA@Fe_3_O_4_ NPs, which visualize a spherical geometry of nanoparticles with streptavidin (SA) proteins (grey colors) wraps Fe_3_O_4_ NPs (black colors) on the outer side. The SA proteins on the Fe_3_O_4_ NPs acts as a chemical linker for binding the nanoparticles on SAM layers *via* the amino coupling reaction and the biotinylated capture antibodies *via* avidin-biotin binding [20,33]. Figure S1b shows the size distribution of the SA@Fe_3_O_4_ NPs, calculated from 100 nanoparticles from the TEM images. The average diameter of the SA@Fe_3_O_4_ NPs is 47 ± 7.7 nm, with 5 nm of the SA proteins and 42 nm of the Fe_3_O_4_ NPs. In addition, Figure S2 shows the XRD patterns of the SA@Fe_3_O_4_ NPs to determine the purity and crystallinity of nanoparticles. The diffraction peaks of the SA@Fe_3_O_4_ NPs at 2θ appear at 43°(400), 57°(511), 63°(440), and 75°(533), which matched with the standard profile JCPDS file no.19-0629 [34]. This result also suggests that the SA proteins do not change the structure and composition of Fe_3_O_4_ NPs because there is no further change in peak positions between the obtained result and the standard profile. Furthermore, the average crystalline size of Fe_3_O_4_ NPs is calculated by using Scherrer equations, which can be expressed as [35]

(1) $$\tau =0.9\frac{\lambda }{\beta \cos\theta }\;,$$

where β is bandwidth spectra, and λ is X-ray source wavelength (0.154 nm). The average calculated crystal size of Fe_3_O_4_ NPs is 16 nm, which is significantly different from the results obtained by the TEM images. Thus, the SA@Fe_3_O_4_ NPs can be considered as polycrystalline rather than single crystals [35].

**Figure 2:**
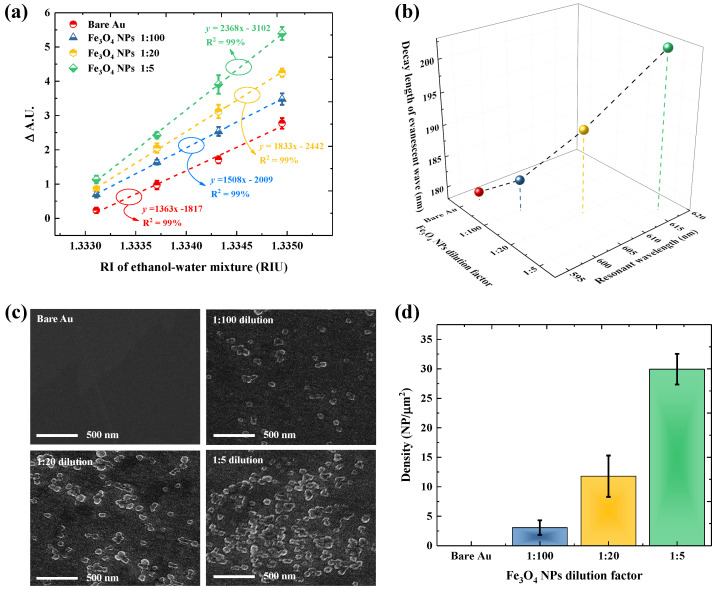
(**a**) Variation of SPR sensitivity in the presence of an ethanol–water mixture by using SA@Fe_3_O_4_ NPs with various dilution factors (1:100, 1:20, and 1:5) and bare Au film. (**b**) Effect of different dilutions of Fe_3_O_4_ NPs on the position of resonant wavelength and the decay length of evanescent field. (**c**) FE-SEM images and (**d**) the calculated density of nanoparticles for Fe_3_O_4_ NPs assembly on Au surface with different dilution factors.

In Figure 2a, we examined the effect of Fe_3_O_4_ NPs toward the SPR sensor sensitivity using different refractive indexes (RI) of ethanol and water mixtures. To ensure maximal sensitivity enhancement, the different dilution factors of Fe_3_O_4_ NPs were considered in SPR experiments. The immobilization of Fe_3_O_4_ NPs on the Au film shows a higher slope compare to the bare Au film. The linearity in the trends is 99% when using bare Au film and Fe_3_O_4_ NPs as the control group. The slope of the calibration curve is correlated with the function of sensitivity [32]; thus, the SPR sensitivity can be further increased by lowering the dilution factor of Fe_3_O_4_ NPs. The greatest sensitivity enhancement can be observed when diluted Fe_3_O_4_ NPs by using a 1:5 dilution factor. The sensitivity of the SPR sensor is improved from 1363/RIU to 2368/RIU where the slope is approximately 1.73-time higher than that of the bare Au chips. Considering the impact of Fe_3_O_4_ NPs on the SPR platform, the sensitivity of Fe_3_O_4_ NPs/SAM/Au film during the subsequent experiment was then expected to be at least 1.73 times higher than that of SAM/Au film.

The impact of Fe_3_O_4_ NPs to enhance SPR sensor sensitivity was evaluated by monitoring the spectra of SPR resonant wavelength at various dilution factors of adsorbed nanoparticles (Figure 2b). A red-shift on the position of resonant wavelength was observed after immobilizing Fe_3_O_4_ NPs on Au films. The shifting of SPR resonant wavelength is directly correlated with the adsorption of SA@Fe_3_O_4_ NPs on the Au surfaces, which was confirmed with an increase in the nanoparticle density visualized by FE-SEM images (Figure 2c). The lower density of nanoparticles corresponding to 1:100 dilution factors of Fe_3_O_4_ NPs resulted in a slight shift of 3 nm on the SPR resonant wavelength. Subsequently, increasing the density of nanoparticles by lowering the dilution factor to 1:5 resulted in a notable wavelength shift until 20 nm. The red-shifted resonant wavelength is correlated with the higher decay length of the evanescent field (*l_d_*) in the vicinity of Au film, which can be expressed as [36]

(2) $$l_{d}=\frac{\lambda_{\mathrm{res}}}{2\pi }\sqrt{\frac{\epsilon_{m}+\epsilon_{d}}{-\epsilon_{d}^{2}}}\;,$$

where ϵ${}_{m}$ and ϵ${}_{d}$ are the metal and dielectric permittivity, respectively, and λ${}_{\mathrm{res}}$ is the resonant wavelength. ϵ${}_{m}$ is defined as a function of wavelength, calculated from Johnson and Christy’s work [37]. The calculated ϵ${}_{m}$ are −9.11, −9.34, −9.87, and −10.62 for the resonant wavelength 596.27 nm, 599.45 nm, 606.50 nm, and 616.35 nm, respectively. The increases in the resonant wavelength cause the permittivity of ϵ${}_{m}$ shiftings towards negative values. According to Equation (2), the higher resonant wavelength and larger magnitude of negative permittivity of ϵ${}_{m}$ are correlated with a longer *l_d_*. Consequently, when using the Fe_3_O_4_ NPs dilution factor of 1:5, *l_d_* of the sensor surface is increased from 179.28 nm to 202.82 nm, which is 1.13-time higher than the bare Au film. Thus, following the increased slope of the calibration curve in Figure 2a, the substantial red-shifted on the resonant wavelength to produce the larger magnitude of the negative permittivity of ϵ${}_{m}$ and the longer *l_d_* can be mainly attributed to Fe_3_O_4_ NPs in terms of enhancing the sensitivity of the SPR sensor.

### 3.2. Selectivity and Sensitivity Evaluation of Antibody with α-syn

**Figure 3:**
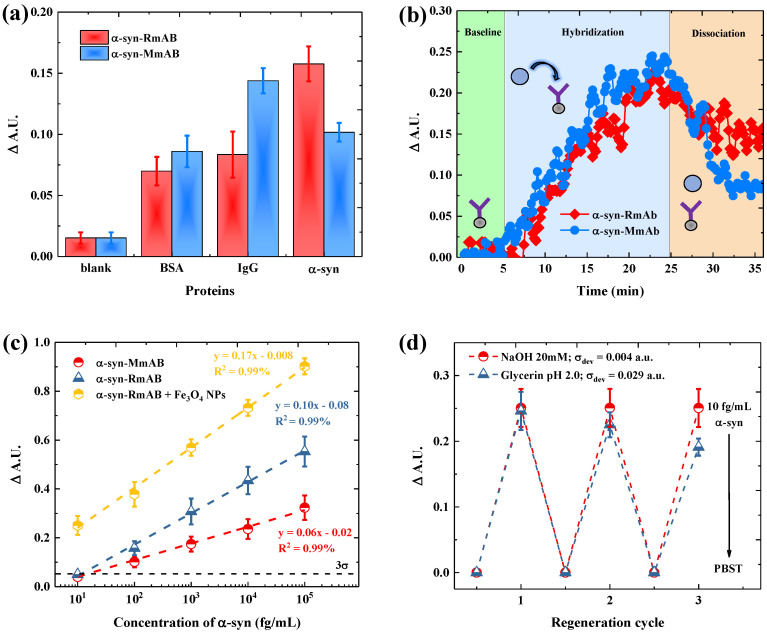
Evaluation of (**a**) selectivity and (**b**) binding affinity of α-syn-RmAb and α-syn-MmAb for detection of 100 fg/mL α-syn. (**c**) Correlation between SPR signal and α-syn concentrations for without Fe_3_O_4_ NPs (α-syn-RmAb or α-syn-MmA) and with Fe_3_O_4_ NPs enhancement. (**d**) Investigation of reproducibility of α-syn-RmAb/Fe_3_O_4_ NPs/SAM/Au sensor chips. The sensor chips was regenerated using a glycerin solution with pH 2.0 (blue dots color) and 20 mM NaOH with pH 9.0 (red dots color). Note: the error bars represent the standard deviation of the three replicated measurements.

The selectivity of monoclonal antibodies-derived from rabbit and mouse hosts against α-syn proteins was evaluated through three injection scenarios, which involve blank, target proteins, and non-specific protein. For the non-specific protein test, BSA and IgG were used as competing proteins because these proteins are highly abundant in human blood [38]. The SPR signal of the α-syn-RmAb and α-syn-MmAb SPR sensors from the three scenarios are given in Figure 3a. For the blank test, the SPR sensor shows a slight increase, where this signal comes from the noise output of the sensor. In the unspecific protein scenario, all competitive proteins increase the SPR signal through non-specific interactions with the SPR sensor surface. For the target scenario, the α-syn-RmAb gives a higher signal of α-syn compare to the signal of BSA and IgG. In contrast, the α-syn-MmAb exhibits a lower signal of α-syn compare to the IgG proteins, while a higher signal of α-syn than the BSA protein. This result implies that IgG may significantly impact the selectivity of α-syn-MmAb when using this antibody in clinical applications. The selectivity coefficient for α-syn-RmAb and α-syn-MmAb, calculated from the results, are summarized in Table 1. The calculated selectivity constants for α-syn-RmAb against BSA and IgG are 2.25 and 1.18, respectively, while the constants for the α-syn-MmAb against BSA and IgG are 1.89 and 0.71, respectively. These results show that the selectivity of rabbit antibody has 1.90 and 1.66 times better than the mouse antibody in reducing the effect of NSB from BSA and IgG, respectively. Thus, the immobilization of α-syn-RmAb on the Au films allows the SPR sensor to enhance α-syn selectively.

Next, the binding capability of the α-syn-RmAb and α-syn-MmAb to capture the α-syn proteins was experimentally assessed and shown in Figure 3b. In this experiment, the target α-syn in PBST buffer was first injected into sensor surface to induce the hybridization process between the α-syn and the capture antibody. During the initial hybridization process, the SPR signal gradually increases due to the binding formation between the α-syn proteins and the capture antibodies. After a specific time, the SPR signal reached a stable baseline, indicating that the hybridization and dissociation of α-syn molecules on the sensor surface occurred at the equilibrium state. After the hybridization process, PBST buffer was flowed onto the sensor surface for 8 min to remove unbound α-syn, which was not strongly captured by the antibody. During this process, the α-syn-RmAb and α-syn-MmAb show dissociation as signal decreasing after wash. Meanwhile, α-syn-RmAb demonstrates a slower dissociation rate than α-syn-MmAb, which was indicated by a slighter decrease in α-syn-RmAb dissociation signal compared to α-syn-MmAb. The slow dissociation rate suggests that the protein forms strong adsorption with the sensor surface. At the same time, the faster dissociation process can be translated as an easier removal by a buffer flow after molecule adsorption on the sensor surface [39,40]. As a result, the signal of α-syn-RmAb (0.15 a.u) shows a higher response than the signal of α-syn-MmAb (0.10 a.u). Thus, the monoclonal antibody derived from a rabbit host has a more robust binding with α-syn molecules than the monoclonal antibody derived from the mouse host.

The series concentration of α-syn using α-syn-RmAb (Figure S3a) and α-syn-MmAb (Figure S3b) was measured based on real-time SPR signals and curve-fitted using linear fitting shown as Figure 3c. The strong binding affinity of α-syn-RmAb causes the SPR signal for each α-syn concentration to be higher than the α-syn-MmAb; thus, resulting in the higher slope trend signal of α-syn for rabbit-type monoclonal antibodies. A correlation coefficient (R^2^) of 99% in the SPR signal trend line of these two antibodies was obtained. The standard deviation of the output SPR signal from the blank measurement (σ) was calculated and multiplied as 3σ. Next, the value of 3σ was divided by the slope of the calibration curve to estimate the minimum concentration value as the limit of detection (LOD = $\frac{3\sigma }{S}$) [41]. Because rabbit antibody produced a higher sensitivity than mouse antibody, the LOD of SPR improved from 75 fg/mL (α-syn-MmAb) to 45 fg/mL (α-syn-RmAb). Thus, this result suggests that the binding affinity plays an essential factor in decreasing the detection limit of the SPR platform. In order to further lower the LOD of the SPR biosensors, the α-syn-RmAb is functionalized with biotins and coupled with the Fe_3_O_4_ NPs via avidin-biotin interaction. As shown in the green dot-line, the combination of high refractive index on Fe_3_O_4_ NPs and strong binding affinity on α-syn-RmAb can further increase the slope of the trend line from 0.10 mL/fg to 0.17 mL/fg. As a result, the LOD of the SPR sensor can be decreased to 5.6 fg/mL. This result demonstrates that the α-syn-RmAb/Fe_3_O_4_ NPs/SAM/Au-based chip is 1.7-time more sensitive than the α-syn-RmAb/SAM/Au-based one, resulting in an 8-time lower LOD. The antibody types, linearity ranges, and LOD values of currently available approaches for α-syn detection are summarized in Table 2. Clearly, the Fe_3_O_4_ NPs plus α-syn-RmAb-assisted SPR sensor has lower LOD than most conventional antibody-based methods.

The reproducibility of the SPR sensor was also of paramount importance to enable the α-syn measurement with a massive and low-cost sample detection. Thus, we evaluated the reproducibility of the α-syn-RmAb/Fe_3_O_4_ NPs/SAM/Au chips toward the use of two regenerating agents (glycerin pH 2.0 and 20 mM NaOH pH 9.0) with a capability to regenerate biosensor surfaces. Figure 3d shows the SPR signal from three continuous injections of α-syn samples with 10 fg/mL concentration onto the targeted sensor surface after a regeneration process involving each one of the regenerating agents. The NaOH regeneration solutions facilitate a similar signal for three cycles with a relative standard deviation ($\sigma_{\mathrm{dev}}$) of 0.004. As reported earlier, the antigen–antibody binding can be reversed since that bond formed from electrostatic forces, which are weak and very dependent on the pH condition of the environment [45,46]. Hence, when NaOH solution changes the pH environment from 7.4 to 9.0, the conformational structure of the antibody can be altered because such a pH range is above the isoelectric point of the antibody (pl ≈ 7–8) [47]. Thereby, the α-syn can be removed from the antibody to reduce the binding sites on the sensor surface. However, when using glycerin pH 2.0, a noticeable attenuation of the signal over three cycles was observed with a noticeable relative standard deviation ($\sigma_{\mathrm{dev}}$ = 0.029). Such an attenuation may be attributed to the extreme pH change which can induce the denaturation of the capture antibody, consequently reducing the bioactivity of antibodies tethered on the surface [47]. Hence, it is critical to consider a suitable regeneration solution to restore the sensor surface with no risk of denaturing the antibody. In this case, the NaOH solution provides good reproducibility on the targeted sensor surface, which leads to a similar level of SPR signal with a low standard deviation signal.

### 3.3. Paired Antibody for Detecting α-Syn in Serum

**Figure 4:**
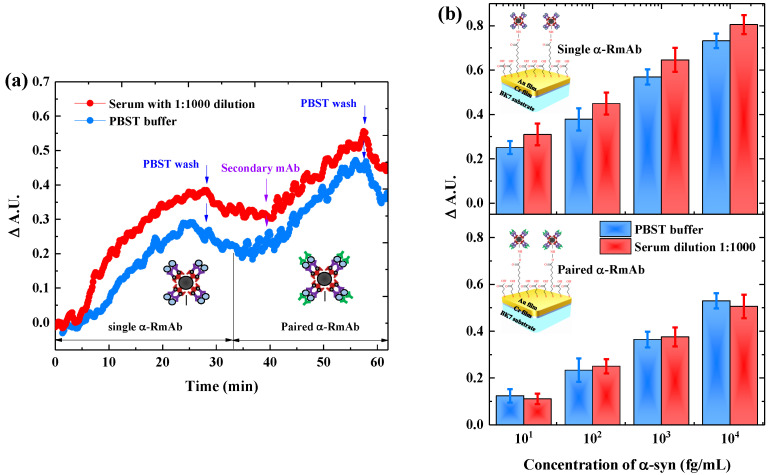
(**a**) Representative SPR sensorgram illustrating the real-time immunoassay of α-syn with a concentration of 10 fg/mL in PBST buffer (blue line) and diluted serum buffer (red line), which involving two detection scenarios: single antibody and paired antibody. (**b**) Correlation of SPR signal with a series concentration of α-syn in PBST buffer (blue color bar) and diluted serum buffer (red color bar) by using the single antibody (upper panel) and the paired antibody (lower panel). The error bar represents the standard deviation of the three replicated measurements.

To test the biosensing practicability, the SPR sensors incorporated with Fe_3_O_4_ NPs and α-syn-RmAb were employed to detect the α-syn proteins in diluted serum samples. As shown in Figure 4a, the single antibody scenario has a significantly higher signal on α-syn detection in serum samples (red line) compared to PBST buffer (blue line). The higher signal of SPR sensorgram in serum compare to PBST buffer can be attributed to the high background signal effect stemmed from the interaction between the non-specific protein and the sensor surface [24,25]. Subsequently, when the secondary antibody was applied after the injection of α-syn, the measured SPR signal from serum is almost similar to the signal from the PBST buffer. This behavior implies that the utilization of secondary mAb can reduce the background signal effect on the SPR sensorgram.

Furthermore, Figure 4b shows the series concentration measured from the SPR signals when used the single antibody (upper panel) and the paired antibody (lower panel). For a single mAb, the detection of each α-syn concentration in serum shows a high signal variation than the PBST buffer, alluding to an NSB effect on the sensor surface. In contrast, the SPR signal of the antibody pair approach to detect α-syn in serum and PBST produces a lower signal variation than that of the single antibody method. To verify the accuracy of these two measurement approaches, the recovery rate must be defined based on the ratio of added and α-syn concentration detected in diluted serum samples. Table 3 shows the calculated values for the recovery rates. The recovery rates for the antibody pair format are 94.5% to 104.3%, revealing the excellent accuracy of the prepared sensors. Note that the rate of 100% means the accuracy of sensor’s to provide an average detection value of α-syn similar to the true value when a sample is added to the complex sample. Meanwhile, the rates for the single antibody format are 324.0% to 525.1% which indicates a significantly higher detectable α-syn compared to the added value of α-syn concentration. This result suggests that the high NSB effect in the single antibody setup causes the indistinguishable concentration between α-syn and un-specific protein. Therefore, the application of Fe_3_O_4_ NPs combined with a pair of α-syn-RmAb for the SPR biosensors provides a promising method in clinical PD diagnosis because it demonstrated high sensitivity and high accuracy in tracing the α-syn until femtogram unit in a serum sample.

## 4. Discussion

In the current study, the development of an SPR biosensor for early diagnosis of PD based on α-syn detection is introduced. This technique considers the immobilization of antigens to receptors, such as aptamers and antibodies, on metal sensing surfaces. Yin et al. [44] demonstrated that the implementation of the SPR platform could be used to quantify the α-syn in cerebrospinal fluid (CSF) liquid biopsy. However, such a detection method has drawbacks; for example, clinically invasive procedures are required in CSF sample collection [13]. As a result, routine assessment of α-syn in CSF is challenging. In this regard, quantifying the level of α-syn in the blood is more realistic for regular clinical testing of PD. For the first time, Sun et al. [48] demonstrated that the conventional SPR technique is very versatile to detect the α-syn in a serum sample until 8 pM (≈1 pg/mL). For initial diagnostic purposes, the concentration of α-syn in clinical human blood, both for PD patients and healthy people, can be shallow until a femtogram unit [14,49]. As a result, this detection limit cannot fulfill the requirement for accurate detection of α-syn in physiological serum. Thus, signal amplification is indispensable for implementing the SPR platform in clinical trials. In addition, the surface specificity of the sensor should be able to withstand the NSB effect of abundant protein in serum buffer during a clinical trial [21]. This is because the binding of non-specific proteins on the sensor surface can cause difficulty to differentiate the signal of specific analytes [21,24]. Therefore, our results demonstrated that the Fe_3_O_4_ NPs incorporated with a pair of antibodies could detect the α-syn in the serum samples in the fashion of high sensitivity and accuracy.

In this study, the different density of Fe_3_O_4_ NPs was applied to maximize sensitivity enhancement in SPR performances. Fe_3_O_4_ NPs are beneficial for increasing the sensitivity of SPR platforms because these nanoparticles have a huge molecular weight and a high refractive index [22]. As shown in Figure 2b, a significant red shifting of SPR resonant wavelength can be produced when a high density of Fe_3_O_4_ NPs is immobilized on the Au surface. The red-shift of the resonant wavelength leads to the ϵ${}_{m}$ of Au metal to become more negative [20,29]. The significant negative of ϵ${}_{m}$ of Au causes the decay length of the evanescent wave to become longer in the direction of surface plasmon propagation [20]. The longer decay length implies a strong electric field in the vicinity of the metal film, resulting in the sensitivity enhancement on the SPR platform [36]. Therefore, as shown in Figure 2a, the high coverage of Au surface by Fe_3_O_4_ NPs can increase the sensitivity of the SPR platform to 1.7 times when the denser nanoparticles are applied. Generally, a standard method to enhance the SPR sensitivity is based on the fabrication of multilayer films on the Au sensing surface. For instance, a multilayer of Au/Ag film, which is a common approach to enhance SPR sensitivity, was reported to increase the SPR sensitivity up to 1.19 times higher than the bare Au film [32]. The multilayer of Au/Ag film can increase SPR sensitivity because Ag films can absorb more visible light than Au, generating higher surface plasmons on metal sensing surfaces [50]. The dielectric film of SiO_2_ coated on the Au film was also reported to increase the SPR sensitivity up to 1.25 times [51]. Moreover, the implementation of 2D material on Au film, such as graphene, can enhance the SPR sensitivity upto 1.34 times higher than the conventional Au film [52]. Based on these comparisons, the utilization of a high refractive index and huge molecular weight of Fe_3_O_4_ NPs clearly shows the potential to improve the sensitivity of Au film compared with the multilayer approach, such as Ag, SiO_2_, and graphene-based materials.

Next, a comparative study of antibodies was performed by comparing the rabbit-type with the mouse-type antibody to determine a suitable bioreceptor for detecting α-syn in the serum sample. As previously reported, cross-reactivity between animal antibodies and non-specific proteins from serum has been shown as one of the influential factors leading to controversial results towards the measurement of serum α-syn levels [13,14,53]. Therefore, it is crucial to observe the binding affinity and selectivity of the antibody before utilizing the SPR platform in a serum test. As shown in Figure 3b, the α-syn-RmAb shows a better affinity than the α-syn-MmAb. The stronger binding affinity of α-syn-RmAb leads to the high selectivity detection on the antigen compared with the α-syn-MmAb (Table 1). The higher binding affinity-selectivity on α-syn-RmAb is because rabbit-type antibodies can recognize epitopes on human antigens that are not immunogenic in mouses, thereby increasing the number of epitopes on rabbit antibodies to identify target antigens [54]. This ability can facilitate the production of antibodies that strongly bind only to the target antigen [54,55]. With the excellent performance of the rabbit-host antibody, the sensitivity of the SPR platform can be further improved by combining the high refractive index of Fe_3_O_4_ NPs and the strong binding affinity of α-syn-RmAb to the Au sensor surface. As shown in Figure 3c, the utilization of α-syn-RmAb/Fe_3_O_4_ NPs/SAM/Au sensor chip enables the SPR platform to detect α-syn protein concentration as low as 5.6 fg/mL. Such detection limit satisfies the requirement to accurately trace an ultra-low level of α-syn in a serum sample until the femtogram unit.

Moreover, in order to test the reusability of the α-syn-RmAb/Fe_3_O_4_ NPs/SAM/Au sensor chip, different regeneration conditions were evaluated. An effective regeneration procedure should remove α-syn bound to α-syn-RmAb, while α-syn-RmAb and Fe_3_O_4_ NPs molecules remain immobilized on the sensor surface. As shown in Figure 3d, excellent repeatability of the SPR signal for detection of 10 fg/mLα-syn was obtained using a NaOH regeneration protocol.

The versatility of α-syn-RmAb/Fe_3_O_4_ NPs/SAM/Au sensor chips in a clinical test was simulated by detecting α-syn in a diluted serum sample. A good accuracy detection was obtained when the paired α-syn-RmAb were used for the SPR experiments. The pair of the α-syn-RmAb condition allows the SPR platform to trace the α-syn in serum samples in a more sensitive and selective manner. During the serum test, the un-specific protein can bind with the sensor surface through electrostatic interaction [24]. Therefore, since the SPR biosensor detects the analyte based on the biomolecular interaction, any NSB that occurred in the proteins on the sensor surface can produce a signal indistinguishable from the desired analyte [20,21]. Adding the secondary antibody after the capture of the analyte in a biofluid can avoid the background effect from the serum [25]. As a result, the quantification of the analyte can be achieved with a low level of non-specific response.

Finally, we demonstrated the use of label-free Fe_3_O_4_ NPs and paired rabbit antibodies for developing a highly sensitive and selective performance of the SPR biosensor in the quantitative detection of serum α-syn, which are a promising biomarker for early diagnosis of PD. The current biosensor has a LOD of 5.6 fg/mL in PBST buffer and diluted serum sample, which is 20,000-times lower than that of commercial ELISA (Table 2). The sensitivity amplification of the SPR biosensor is mainly based on the direct immobilization of Fe_3_O_4_ NPs. The assembly of the high refractive index Fe_3_O_4_ NPs on the sensor surface allows us to modulate the ϵ${}_{m}$ of the Au surface, which improve the LOD of the SPR sensor until 8-times lower than the conventional Au films. This approach also enables the SPR platform to detect the low α-syn concentration with a simpler procedure, low-cost, and rapid measurement. Furthermore, this study also introduced the versatility of rabbit host antibodies in the quantification of α-syn. Rabbit monoclonal antibodies show a potential to be used in the diagnosis of PD due to the higher affinity and selectivity binding of the α-syn-RmAb compare to the α-syn-MmAb. Remarkably, the combination of the label-free Fe_3_O_4_ NPs and the α-syn-RmAb in the SPR sensor chips shows a sensitive, selective, and regenerable performance, making this platform a potential alternative for detecting α-syn clinically with an affordable point-of-care test. To the best of our knowledge, this is the first study to introduce the combination of the label-free Fe_3_O_4_ NPs and the rabbit monoclonal antibodies to detect α-syn in blood samples. Thus, this approach represents a novel technique to detect α-syn in blood-based samples and has an extraordinary opportunity for the development of biosensing platforms for the early diagnosis of PD. However, this study has limitations in real clinical practices. Our study was performed with a serum diluted by 1000-times, consequently reducing the required level of detection of serum α-syn from femtogram level to attogram level. In this regard, two approaches can be considered to overcome the limitations of the work: (1) improving the specificity detection of the current SPR platform for detecting α-syn in undiluted serum samples or (2) amplify the sensitivity of the current SPR platform to the level of attogram detection. The first approach can permit the current LOD of the SPR platform to detect α-syn in clinical serum samples, which can be obtained in several ways. For instance, the use of magnetic properties in Fe_3_O_4_ NPs are capable to separate the target antigen from the un-specific protein in undiluted serum samples, which can effectively reduce the background interference effect [22,56]. This process also allows Fe_3_O_4_ NPs to enrich the target analyte, which leads to sensitivity enhancement until 10${}^{2}$-fold higher than the conventional Au films due to the significant changes in the binding-induced refractive index [28,56]. Additionally, coating the sensor surface with antifouling materials, such as polyethylene glycol (PEG), can significantly upgrade the selectivity of the SPR sensing surface in the undiluted serum samples by up to 99% [21]. The key factors of PEG layers in reducing the NSB effect reside on the steric–entropy barrier characteristic and a high degree of hydration to repel the interaction of protein on the sensor surface [57]. Furthermore, the second approach can enable the SPR sensor to detect very low α-syn concentration when using the dilution serum to reduce the NSB effect. Au nanostructures (such as AuNPs and Au nanorods (AuNRs)) have been reported as one of the best candidates for improving the sensitivity of SPR sensors [22]. For instance, using label-tag AuNPs on the SPR gold surface can lead to strong optical coupling of incident light to resonance, increasing the SPR sensitivity by up to 10${}^{2}$-fold [58]. Additionally, the use of label-tag AuNRs can enhance the SPR sensitivity until 10${}^{5}$-fold higher than the Au film, resulting in the LOD of SPR biosensor down to 10 aM for the detection of miRNA biomolecules [59]. AuNRs show a stronger enhancer factors than AuNPs because of having a two plasmon resonance wavelength in the longitudinal and transverse modes [22]. Consequently, AuNRs have a much larger wavelength range of plasmon resonance than the AuNPs, so the AuNRs own a more vigorous electric field intensity than the AuNPs [22,28]. Taking these enhancement sensitivity factors into account, the use of AuNRs could be more advantageous to improve the sensitivity of the current SPR platform to allow detection of α-syn at the attogram level quantification. Therefore, further studies on these two approaches are needed to optimize the detection performance of current SPR platforms, so this new technique becomes effective for detecting serum α-syn in clinical applications.

## 5. Conclusions

A highly sensitive and selective SPR biosensor based on label-free Fe_3_O_4_ NPs and α-syn-RmAb has been successfully developed to detect α-syn in a serum sample. The Fe_3_O_4_ NPs density on the sensor surface was modulated by varying the dilution factor of nanoparticles. Increasing the Fe_3_O_4_ NPs density on the Au film leads to the more negatively permittivity of metal, cause the enhancement of decay length on the metal film. As a result, the sensitivity of the SPR platform was amplified markedly until 1.7-time by immobilizing the high density of Fe_3_O_4_ NPs on the Au sensor surface. Moreover, the binding performance of the antibody derived from the rabbit and mouse was investigated to determine a suitable bioreceptor for a serum test. We found that the α-syn-RmAb is preferable over α-syn-MmAb, due to the higher binding affinity and better selectivity on monoclonal antibody-derived from rabbits hosts compare with the mouse host. As a result, the combination of high-density Fe_3_O_4_ NPs and α-syn-RmAb allowed the SPR platform to detect α-syn as low as 5.6 fg/mL, which is 8-time lower LOD than that of the bare Au film. The sensor surface also shows a regenerable feature up to three-cycle measurement by using NaOH solutions. In serum tests, the α-syn-RmAb/ Fe_3_O_4_ NPs/SAM/Au sensor chips show an excellent detection performance with an accuracy recovery detection in the range from 94.5% to 104.3% by using a pair of α-syn-RmAb format. Thus, the improved platform demonstrates high sensitivity, good reproducibility, and high accuracy, making it an excellent alternative for detecting α-syn clinically. However, given that the α-syn detection in this study was performed at a high dilution factor, further improvements in sensing specificity are needed to enable high-throughput clinical PD diagnosis.

## Figures and Tables

**Table 1:** Selectivity coefficient of the α-syn-RmAb and α-syn-MmAb on SPR sensor.

Proteins	Δ A.U.		Selectivity Coefficient	
	α-syn-RmAb	α-syn-MmAb	α-syn-RmAb	α-syn-MmAb
α-syn	0.16 ± 0.02	0.10 ± 0.01	—	—
BSA	0.07 ± 0.01	0.09 ± 0.01	2.25	1.18
IgG	0.08 ± 0.02	0.14 ± 0.01	1.89	0.71

**Table 2:** Comparison with other analytical techniques based on the antibody for α-syn detection.

Technique	Capture Antibody	Linearity Range (pg/mL)	LOD (pg/mL)	Ref
Electrochemical	mouse mAb	(10–1000) × $10^{3}$	1130	[17]
sFIDA	mouse 4B12 mAb	(3.60–26) × $10^{3}$	980	[18]
ELISA	mouse 4B12 mAb	(0.20–3.20) × $10^{3}$	114	[42]
Neurobiosensors	mouse mAb	4–2000	0.1350	[19]
IMR	mouse sc-12767 mAb	(0.31–31)× $10^{3}$	0.0003	[43]
SPR	rabbit MJF-R13 mAb	0.10–10	0.0700	[44]
	rabbit Ab138501 mAb	0.01–100	0.0056	This work

**Table 3:** Recovery rate of the single α-syn-RmAb and the paired α-syn-RmAb on SPR sensor.

Sample	Added α-syn	Found (fg/mL)		Recovery Rate (%)	
	(fg/mL)	Single mAb	Paired mAb	Single mAb	Paired mAb
1	10	36.6	9.9	366.2	99.4
2	100	324.0	104.3	324.0	104.3
3	1000	5251.5	1032.5	525.1	103.2
4	10,000	49,107.1	9455.6	491.1	94.5

## Data Availability

Not applicable.

## Supplementary Materials

The following are available online at https://www.mdpi.com/article/10.3390/bios11100402/s1. Figure S1: Fe_3_O_4_ NPs characterization. (a) TEM images of SA@ Fe_3_O_4_ NPs on a glass substrate with scale bar 20 nm. Two arrows with green and purple color denotes SA proteins (the grey-color dots) and Fe_3_O_4_ NPs (the black-color dots), respectively. (b) Histogram of SA@Fe_3_O_4_ NPs size distributions and their Gaussian fitting shows the diameter of SA@Fe_3_O_4_ NPs ranging from 34 nm to 75 nm with an average nanoparticle diameter of 47 ± 7.7 nm. Figure S2: XRD patterns for the prepared SA@Fe_3_O_4_ NPs (the red-color line) and the reference of the standard magnetite from JCPDS datasheet no. 19-0629 (the blue-color line). Figure S3: Real-time SPR signals from the detection of α-syn concentration from 100 fg/mL to 100 pg/mL by using (a) monoclonal antibody derived from rabbit host (α-syn-RmAb) and (b) derived from mouse host (α-syn-MmAb).
