# Supplementary material for: Enhanced Plasmonic Biosensor Utilizing Paired Antibody and Label-Free Fe3O4 Nanoparticles for Highly Sensitive and Selective Detection of Parkinson’s α-Synuclein in Serum
Source: Biosensors (Basel). 2021 Oct 18;11(10):402. doi: 10.3390/bios11100402 (PMC8534275; doi:10.3390/bios11100402)
Supplement: Supplementary file 1 [file biosensors-11-00402-s001.zip › biosensors-1407316-supplementary.pdf]

biosensors

Supplementary Materials

Enhanced Plasmonic Biosensor Utilizing Paired Antibody and Label-free Fe_3O_4 Nanoparticles for Highly Sensitive and Selective Detection of Parkinson's α-Synuclein in Serum

Samuel Husin Surya Mandala [1,‡], Tai-Jan Liu [2,‡], Chiung-Mei Chen [3,‡], Kuo-Kang Liu [4], Mochamad Januar [1], Ying-Feng Chang [5], Chao-Sung Lai [1,5,6,7,8], Kuo-Hsuan Chang [9], and Kuo-Chen Liu [1,8,10*]

[1] Department of Electronic Engineering, Chang Gung University, Taoyuan 33302, Taiwan; samuelhusin31@gmail.com (S.H.S.M); moch.januar@outlook.com (M.J); cslai@mail.cgu.edu.tw (C.-S.L)
[2] Department of Traditional Chinese Medicine, Chang Gung Memorial Hospital, Keelung 204, Taiwan; sarah78912@hotmail.com (T.-J.L)
[3] Department of Neurology, Chang Gung Memorial Hospital, Chang Gung University College of Medicine, Taoyuan 33302, Taiwan; cmchen@cgmh.org.tw (C.-M.C)
[4] School of Engineering, University of Warwick, Coventry CV4 7AL, United Kingdom; I.K.Liu@warwick.ac.uk (K.-K.L)
[5] Artificial Intelligence Research Center, Chang Gung University, Taoyuan 33302, Taiwan; yfchang@mail.cgu.edu.tw (Y.-F.C)
[6] Biosensor Group, Biomedical Engineering Research Center, Chang Gung University, Taoyuan 33302, Taiwan
[7] Department of Nephrology, Chang Gung Memorial Hospital, Linkou, New Taipei City 33305, Taiwan
[8] Department of Materials Engineering, Ming Chi University of Technology, New Taipei City 24301, Taiwan
[9] Department of Neurology, Chang Gung Memorial Hospital Linkou Medical Center and College of Medicine, Chang Gung University, Linkou 33305, Taiwan; gophy5128@cgmh.org.tw (K.-H.C)
[10] Division of Pediatric Infectious Disease, Department of Pediatrics, Chang Gung Memorial Hospital, Linkou 33305, Taiwan
* Correspondence: jacobliu@mail.cgu.edu.tw (K.-C.L.);
‡ The three authors contributed equally to this work

S1. Characterization of Fe_3O_4 NPs

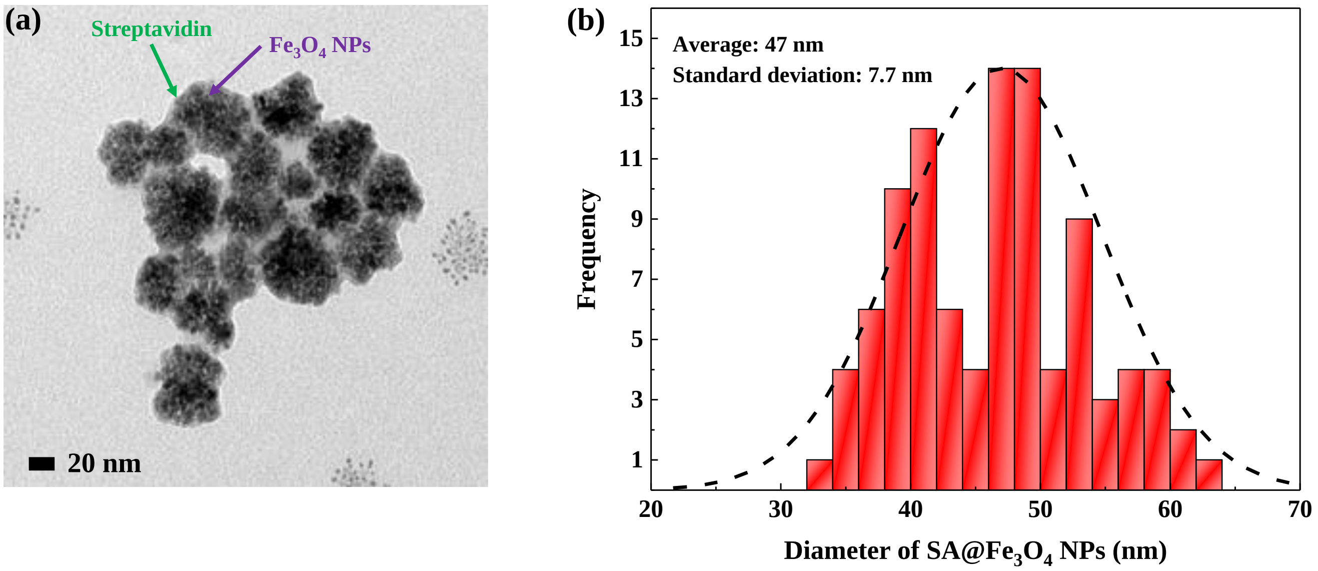

Figure S1. Fe_3O_4 NPs characterization. (a) TEM image of SA@ Fe_3O_4 NPs on a glass substrate with scale bar 20 nm. Two arrows with green and purple color denotes SA proteins (the grey dots) and Fe_3O_4 NPs (the black dots), respectively. (b) Histogram of SA@ Fe_3O_4 NPs size distributions and their Gaussian fitting shows the diameter of SA@ Fe_3O_4 NPs ranging from 34 nm to 75 nm with an average nanoparticle diameter of 47 ± 7.7 nm.

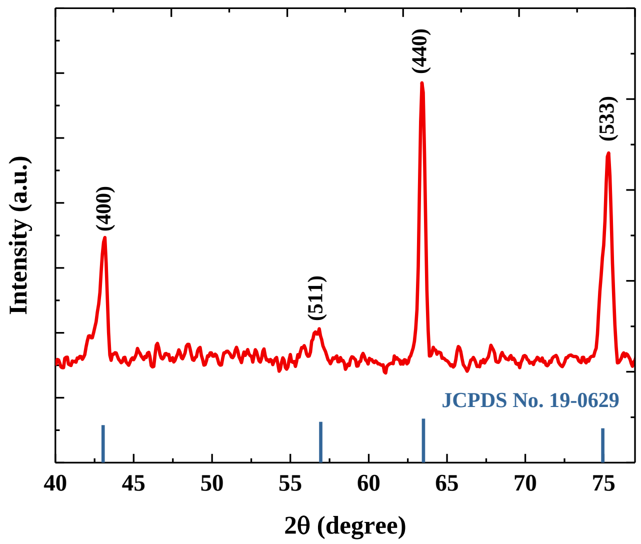

Figure S2. XRD patterns for the prepared SA@Fe_3O_4 NPs (the red-color line) and the reference of the standard magnetite from JCPDS datasheet no. 19-0629 (the blue-color line).

S2. Binding performance of the α-syn-RmAb and α-syn-MmAb

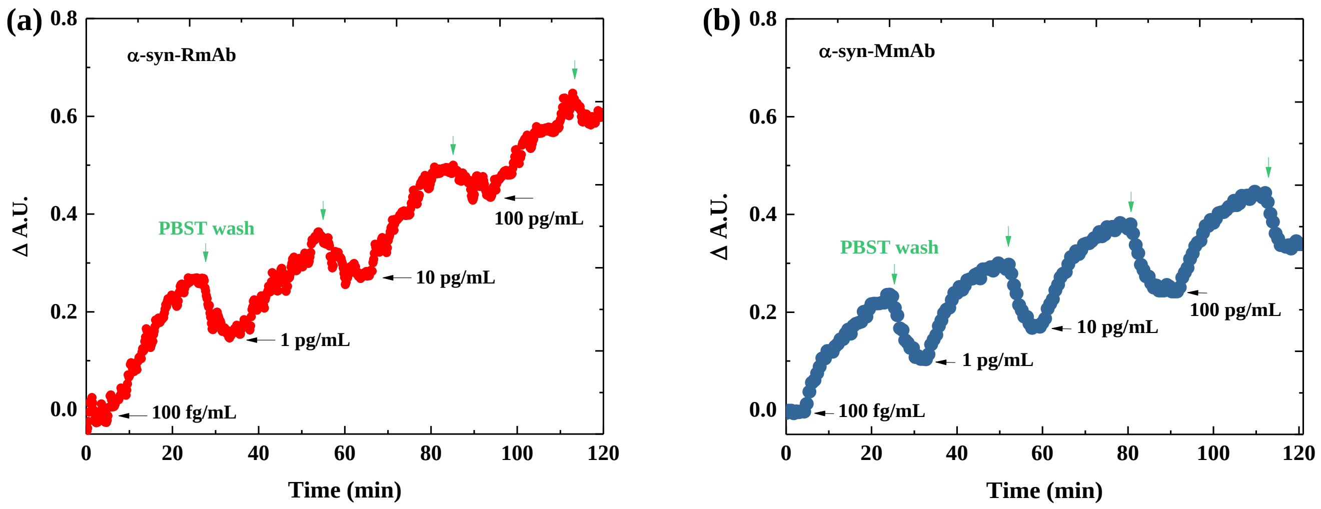

Figure S3. Real-time SPR signals from the detection of α-syn concentration from 100 fg/mL to 100 pg/mL by using (a) monoclonal antibody derived from rabbit host (α-syn-RmAb) and (b) derived from mouse host (α-syn-MmAb).
